# Modelling the structural disorder in trigonal-prismatic coordinated transition metal dichalcogenides

**DOI:** 10.1107/S1600576723001589

**Published:** 2023-03-30

**Authors:** Federica Ursi, Simone Virga, Candida Pipitone, Alessandra Sanson, Alessandro Longo, Francesco Giannici, Antonino Martorana

**Affiliations:** a Dipartimento di Fisica e Chimica, Università di Palermo, Palermo, I-90128, Italy; b Istituto di Scienza e Tecnologia dei Nateriali Ceramici, Consiglio Nazionale Delle Ricerche, Via Granarolo 64, Faenza, I-48018, Italy; c European Synchrotron Radiation Facility, Grenoble, Cedex 9, France; d Istituto per lo Studio dei Materiali Nanostrutturati, Consiglio Nazionale Delle Ricerche, Palermo, I-90146, Italy; HPSTAR and Harbin Institute of Technology, People’s Republic of China

**Keywords:** chalcogenides, disorder, stochastic modelling, X-ray diffraction

## Abstract

Trigonal-prismatic coordinated transition metal dichalcogenides (TMDCs) are formed from stacked (chalcogen)–(transition metal)–(chalcogen) triple layers, where the chemical bond is covalent within the triple layers and van der Waals (vdW) forces are effective between the layers. Bonding is at the origin of the great interest in these compounds, which are used as 2D materials in applications such as catalysis, electronics, photoelectronics, sensors, batteries and thermoelectricity. This paper addresses the issue of modelling the structural disorder in multilayer TMDCs. The structural model takes into account stacking faults, correlated displacement of atoms and average crystallite size/shape, and is assessed by simulation of the X-ray diffraction pattern and fitting to the experimental data relative to a powdered sample of MoS_2_ exfoliated and restacked via lithiation. From fitting, an average crystallite size of about 50 Å, nearly spherical crystallites and a definite probability of deviation from the fully eclipsed atomic arrangement present in the ordered structure are determined. The increased interlayer distance and correlated intralayer and interlayer atomic displacement are attributed to the presence of lithium intercalated in the vdW gap between triple layers (Li/Mo molar ratio of about 0.06). The model holds for the whole class of trigonal-prismatic coordinated TMDCs, and is suitably flexible to take into account different preparation routes.

## Abstract 2

The structural disorder in trigonal-prismatic coordinated transition metal layered dichalcogenides is investigated. The structural model taking into account stacking faults, correlated displacement of atoms and average crystallite size is assessed by fitting to the X-ray diffraction pattern of an exfoliated–restacked MoS_2_ sample.

## 1. Introduction

The literature on the structure of disordered systems is rooted in the early decades of the last century (Wilson, 1942 ▸; Jagodzinski, 1949 ▸; Hosemann, 1951 ▸), but has been enhanced greatly in the recent time frame by the recognition of the peculiar functional properties deriving from electronic confinement in 2D materials (Ranjan *et al.*, 2022 ▸). This renewed interest started with the pioneering paper on graphene by Novoselov *et al.* (2004 ▸), which was followed by huge scientific productivity, but involves today a plethora of different compounds characterized by a strong bond inside 2D structural units and by a weak interaction between them. Among this latter class, triple-layer transition metal dichalcogenides (TMDCs), composed of a transition metal (TM) sheet (mostly from groups 4–7 and in particular Ti, Nb, Ta, Mo, W, Re) sandwiched between two chalcogen layers (S, Se, Te) and kept bound by covalent metal–chalcogen interactions, are involved in applications including catalysis, electronics, photoelectronics, sensors, batteries and thermoelectricity (Chhowalla *et al.*, 2013 ▸; Li *et al.*, 2020 ▸; Wang *et al.*, 2022 ▸). The 2D nature of these compounds can be exploited by suitable ways of preparing monolayer films, obtained by top-down procedures, such as mechanical or liquid-phase exfoliation, or by bottom-up techniques, such as chemical or physical vapour deposition.

The structure and defectivity of mono- or few-layer TMDC films have been mostly investigated by spectroscopy, microscopy and computational approaches (Xu *et al.*, 2018 ▸; Xiong *et al.*, 2020 ▸; Upadhyay *et al.*, 2021 ▸; Bertoldo *et al.*, 2021 ▸; Cowie *et al.*, 2022 ▸). A few papers report structural analyses carried out by simulation of the total X-ray diffraction (XRD) powder pattern (Yang *et al.*, 1991 ▸; Yang & Frindt, 1996 ▸); in particular, Pakharukova *et al.* (2020 ▸) stress that the presence of a noticeable concentration of structural defects determines a significant size underestimation with respect to electron microscopy approaches.

Multilayer TMDCs can be obtained by various bottom-up procedures, mostly chemical (Frey *et al.*, 1999 ▸; Niefind *et al.*, 2015 ▸; Bekx-Schürmann *et al.*, 2020 ▸; Sanikop & Sudakar, 2020 ▸), but also by top-down routes such as mechanical grinding of bulk samples or exfoliation–restacking. In the latter technique, the exfoliation step can be achieved by mechanical processing (Novoselov *et al.*, 2005 ▸) or by intercalation of various organic or inorganic moieties in the van der Waals (vdW) gap between the 2D TMDC bricks followed by suspension of the easily exfoliated phase in a suitable solvent; the successive restacking step can be achieved by drying the suspension and eventually baking (Yang & Frindt, 1996 ▸). The outcome of most of these procedures is a disordered stacking of the 2D metal–chalcogen slabs. Electrochemical (Sanikop *et al.*, 2020 ▸) or vacuum heating deintercalation (Wan *et al.*, 2017 ▸) can also be a source of stacking disorder. A recent comprehensive review of the synthesis and characterization of nanostructured TMDCs is given by Phalswal *et al.* (2022 ▸).

Spectroscopy and microscopy techniques are exploited for the characterization of multilayer TMDCs, but several studies involving the careful analysis of diffraction patterns are also documented and give valuable and detailed information about the actual atomic arrangement of the investigated samples. We cite in particular (i) the early papers by Frindt and coworkers (Joensen *et al.*, 1986 ▸, 1987 ▸; Yang *et al.*, 1991 ▸; Gordon *et al.*, 2002 ▸), which report extended X-ray absorption fine structure experimental analyses and numerical simulations of the XRD patterns of single-layer, few-layer and restacked MoS_2_ and WS_2_ samples; and (ii) the atomic pair distribution function (PDF) analyses performed on MoS_2_ and WS_2_ by different groups over a wide time span (Petkov *et al.*, 2000 ▸; Mangelsen *et al.*, 2019 ▸; Bekx-Schürmann *et al.*, 2020 ▸; Chithaiah *et al.*, 2020 ▸; Kisała *et al.*, 2022 ▸; Sreedhara *et al.*, 2022 ▸). An overview of this literature shows that, depending on the preparation routes and the exploited experimental techniques, different atomic arrangements are described: (i) metastable octahedral coordination in monolayers, whereas the bulk specimens are trigonal-prismatic; (ii) fullerene-like or multiwalled nanotubes keeping the local sheet arrangement of planar structures; (iii) turbostratic stacking; (iv) zigzag chains of metal–metal bonds; (v) stacking faults. A mixture of different sources of disorder is often allowed.

In this paper we focus on trigonal-prismatic coordination in multilayered TMDCs, using the intensively investigated compounds MoS_2_ and WS_2_. The approach consists of the formulation of a general structural model, described in Section 2, accounting for structural disorder and size distribution; this is followed by the simulation of the diffraction pattern and its fitting to experimental data (in Section 4). As an experimental counterpart (details on sample preparation and characterization techniques are given in Section 3), we make reference to a specific sample of MoS_2_ obtained by di­butyl lithium reaction with ground commercial MoS_2_, followed by exfoliation–restacking of 2D triple-layer units. In Section 5 the significance of some features of the model is discussed; Sections 6 and 7 deal with structural aspects described in the literature, and in particular with turbostratic (in Section 6) and octahedral arrangement (in Section 7).

## 2. The model

The description of the structural model of MoS_2_ encompasses (i) the stacking sequences of the S–Mo–S sandwiches; (ii) the uncertainty in the relative position of atoms belonging to the same layer and to different *n*th-neighbouring layers; (iii) the shape and size distribution of the crystallites; (iv) simulation and fitting of the powder pattern.

### 2.1. Stacking sequences

**Figure 1:**
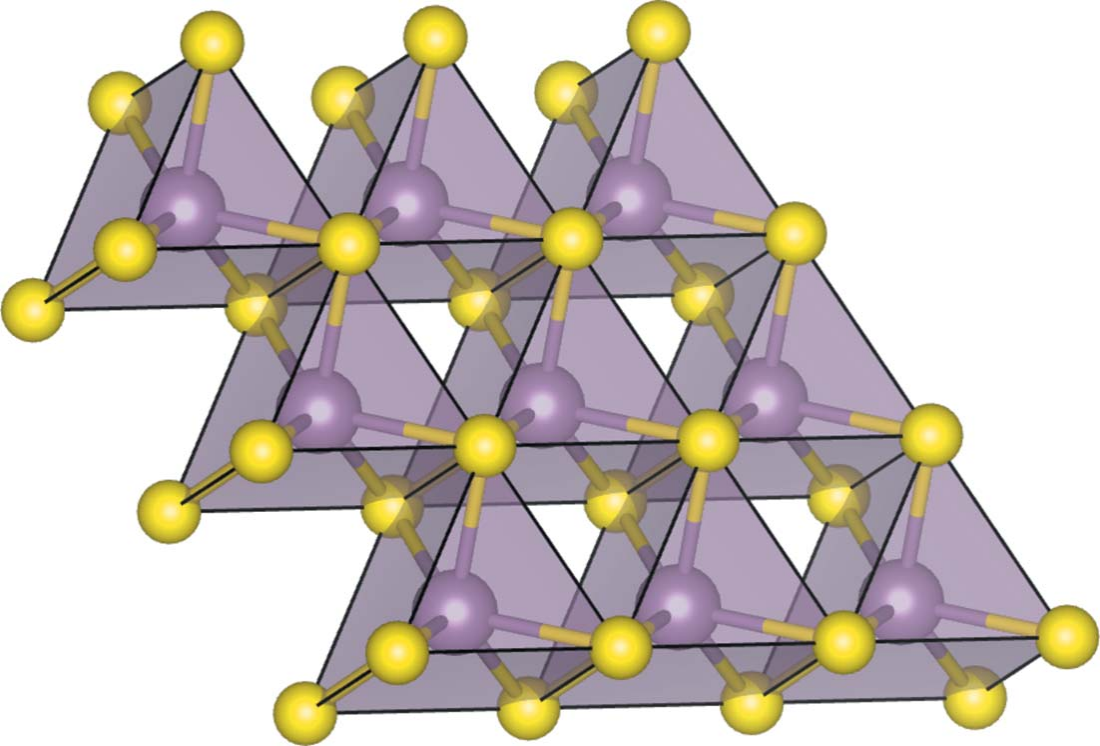
2D network of edge-sharing MoS_6_ trigonal prisms.

The model assumes that the structural units of MoS_2_ are S–Mo–S sandwiches stacked in disordered sequences, fulfilling the constraint of close packing between facing sulfur layers and keeping the same average spacing between the triple layers, irrespective of the close packing sequence. The coordination polyhedra are edge-sharing MoS_6_ trigonal prisms, as depicted in Fig. 1 ▸. The stacking of these units gives rise to the 3D MoS_2_ structure.

**Figure 2:**
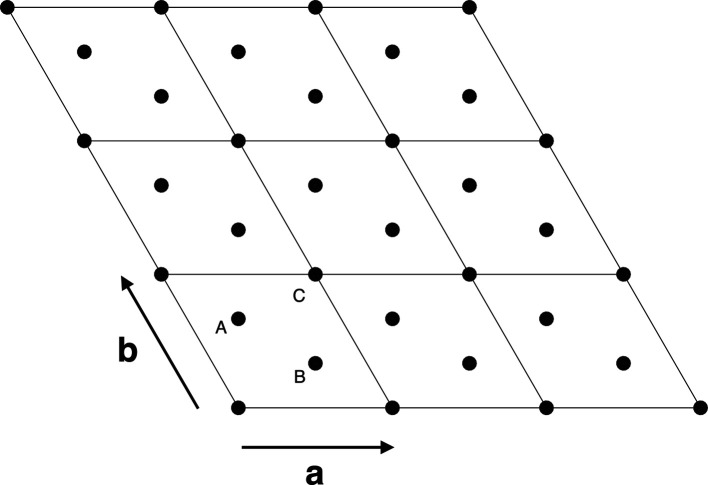
(**a**, **b**) section of the MoS_2_ hexagonal frame. The allowed Mo and S projections onto the (**a**, **b**) plane are indicated; for the sake of clarity, the metal sites are cited in the text with lower-case lettering.

Fig. 2 ▸ shows the available positions of sulfur and molybdenum with respect to the (*a*, *b*) axes of the reference hexagonal frame. According to the trigonal-prismatic coordination, the sulfur–molybdenum–sulfur atomic layers can, respectively, occupy, with reference to the cell depicted in Fig. 2 ▸, the positions AbA, AcA, BaB, BcB, CaC, CbC, where the capital letters refer to sulfur and lower-case letters to molybdenum. The ideal bulk structure of MoS_2_ (*P*63/*mmc*) corresponds to the sequence AbA–BaB–⋯ [*i.e.* AA′ sequence, according to the formalism corresponding to fully eclipsed atoms (Constantinescu *et al.*, 2013 ▸; He *et al.*, 2014 ▸; Bampoulis *et al.*, 2018 ▸)], but the AbA–BcB–CaC⋯ polytype [*i.e.* space group *R*3*m* (Schönfeld *et al.*, 1983 ▸)], with partially staggered atoms [*i.e.* AB sequence (He *et al.*, 2014 ▸)], is also described.

Because of the vdW weak interaction between neighbouring layers, different synthetic routes result in similar diffraction patterns characterized by faulted stacking of the chalcogen–metal–chalcogen layers. This disorder has been associated with modification of electronic structure and with fine-tuning of functional properties in a number of published papers.

The modelling of stacking faults was carried out within the formalism of Kakinoki & Komura (1965 ▸), *i.e.* considering the stacking of the triple layers as a Markov chain, where the *n*th step influences in a probabilistic sense the (*n*+1)-th event. Making reference to the position of metals and chalcogens drawn in Fig. 2 ▸, the relevant stochastic matrix is defined as

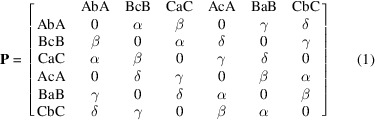

where 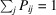 , so that the four parameters of equation (1) are constrained with, for instance, 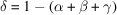 . The case γ = 1 corresponds to the ordered structure in the *P*63/*mmc* space group.

The intensity equation worked out by Kakinoki & Komura (1965 ▸) was modified to get the spherical average in the reciprocal space suitable for fitting to the experimental data:

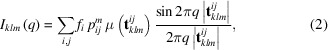

where the sums over *i* and *j* run over the six events heading the rows and columns of equation (1); 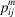 are the elements of the *m*th power of 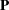 , 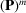 , which is still stochastic; 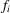 are the frequencies of existence of the *i*th event, bound by the linear and homogeneous system 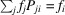 ; by symmetry, 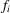 = 1/6. 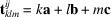 are the distance vectors between an atom belonging to the *i*th-type triple layer and an atom belonging to the *j*th, *m* steps ahead in the **c** direction; then, the index *m* is relative to the vertical component of the interatomic vector, while the indexes *kl* scan the horizontal component parallel to the (**a**, **b**) plane. 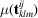 is the multiplicity of the 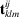 inter­atomic distance, calculated as 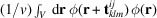 (Gilbert, 2008 ▸). *v* is the volume of the triple-layer unit cell, and *V* is the volume common to the shape function 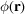 [ 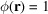 inside the crystallite, 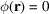 outside], taking into account that there are twice as many chalcogen as metal atoms. 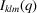 represents the contribution to the total intensity resulting from the interference, averaged over all the allowed *i*, *j* pairs of layers, of two atoms separated by the distance vector 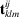 . The overall scattering intensity (see below) is given by the sum of all terms like equation (2), weighted by the respective atomic factors of the atoms separated by the 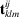 interatomic distance.

### 2.2. Size/shape

The possible shape anisotropy of the crystallites was taken into account assuming that the shape function 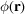 is relative to a spheroid (Gilbert, 2008 ▸), that is, an ellipsoid with two different axes, 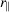 parallel to the (**a**, **b**) plane and 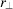 parallel to **c**. The model also allows for a size distribution, assuming that all the crystallites have the same shape, except for a proportionality factor; it is assumed that the size distribution is governed by a simple exponential law, so that the distance multiplicity is given by the weighted sum of terms corresponding to spheroidal crystallites from a minimum to a maximum size:

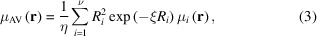

where 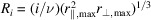 , ξ is a fitting parameter controlling the shape of the exponential distribution, 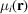 is the distance multiplicity of the *i*th allowed spheroid size, corresponding to 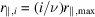 and 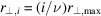 , and η = 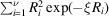 ; ν is the number of allowed spheroid sizes, and **r** represents the generic distance between atoms. Explicit mathematical forms for **r** and μ*_i_* are given above, in Section 2.1.

Size and stacking sequences are decoupled, that is, it is assumed that the probabilities defined in equation (2) hold on the average for the whole sample, no matter what the size of the crystallites.

### 2.3. Uncertainty in the relative position of atoms

As will be shown in the next section, the experimental evidence seems to suggest that the relative position of atoms is affected by an uncertainty and that this uncertainty propagates as a function of the distance; then, a correlated displace­ment of atoms from the ideal crystallographic positions occurs. The formalism initially worked out by Hosemann (1951 ▸) for polymeric materials and called ‘ideal paracrystal’ was also applied to inorganic samples (Hosemann & Bagchi, 1952 ▸; Ruland, 1965 ▸). According to the theory, the mutual position of atoms is not given by a delta function, but rather by a Gaussian distribution, the width of which increases linearly with the distance between atoms:

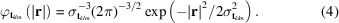




The model allows for the possibility that the intralayer distribution could be different from the interlayer one:

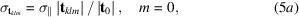




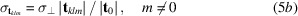

where |**t**_0_| is a unit reference length.

The Fourier transform of (4),

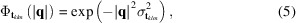

enters as a multiplication factor in the intensity terms defined in equation (2) (Longo & Martorana, 2008 ▸; Longo *et al.*, 2014 ▸, 2020 ▸).

### 2.4. The overall model intensity *I*(*q*)

The assembly of all the features described above yields the total model intensity:

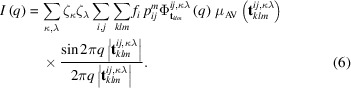




The sum over κ,λ runs over the different pairs of atoms, Mo–Mo, S–S and S–Mo, taking into account the 2:1 stoichiometric ratio between sulfur and molybdenum; to avoid confusion with frequencies, the scattering factors are indicated with ζ instead of the traditional *f*.

### 2.5. Powder pattern simulation and fitting

The accelerated sum of the Debye series was used by Cervellino *et al.* (2006 ▸) to carry out the simulation of the powder pattern of ordered crystallites. In this paper we adopted their approach, but with the addition of a necessary modification to deal with the case of 1D disorder. Thus, the accelerated convergence procedure of Cervellino and coworkers was ‘sliced’ in 2D sums of the type in equation (2), each one corresponding to a fixed *m*-index value or, in other words, to a given height jump. Despite this unavoidable complication, the CPU time was still definitely viable. The fitting procedure was carried out with the package *MINUIT* (James & Roos, 1975 ▸).

## 3. Experimental

A commercial MoS_2_ sample (Alfa Aesar, 98%, −325 mesh) was milled in a high-energy planetary mill (Fritsch, Poulverisette 6) using zirconia beads as grinding media and deionized water (Milli-Q) as solvent. The milling cycle included 36 repetitions of 30 min for a total milling time of 18 h. The milling speed was kept at 400 r min^−1^, using a powder-to-beads volume ratio of 1:15. The obtained powder was centrifuged at 6000 r min^−1^ for 20 min and vacuum dried at 160°C. The dried powder was sieved at 100 µm to obtain the ground–dried sample. This ground–dried sample did not show the presence of secondary phases, while a definite reduction of the preferred orientation present in the fresh sample and a crystallite size reduction of about 50%, leading to an average size of 40 nm, were observed by XRD. We then reacted 8 mmol of the sample with 15 ml of a 1.6 *M* solution of *n*-butyl­lithium in hexane under a nitro­gen atmosphere at room temperature for 1 week (molar ratio Li/Mo = 3), and the li­thia­ted product was washed with hexane and dried under vacuum. The lithium content was determined with flame emission spectroscopy both directly on the solid (dissolved with hot concentrated sulfuric acid and hydrogen peroxide) and by difference from the washing solution, giving an effective Li/Mo molar ratio of about 0.06 in the li­thia­ted solid.

To eliminate residual preferential orientations, which mostly enhance the 00*l* reflections, the sample was mixed with amorphous silica powder. Different blendings were tested, until the diffraction intensity ratios between the 00*l* and the *hkl* reflections reached a steady value at a weight dilution of 1:10. The XRD pattern was recorded with a Rigaku Miniflex 600 diffractometer equipped with an Si strip detector using Ni-filtered Cu *K*α radiation and an incident slit of 1.25° on the primary beam.

## 4. Results

**Figure 3:**
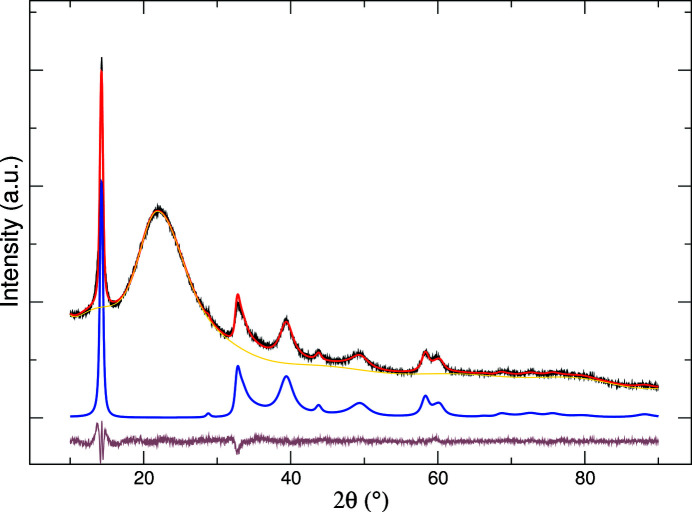
Fitting of the structural model to the XRD data of the exfoliated–restacked MoS_2_ sample. Experimental, black; calculated, red; background, yellow; model, blue; residual, brown. The halo at 22° 2θ is due to the amorphous silica diluent.

Fig. 3 ▸ shows the result of the fitting of the model to the experimental pattern, and Table 1 ▸ reports the values and the uncertainties of the structural parameters described in Section 2.

A non-negligible expansion of the interlayer spacing with respect to bulk MoS_2_ [amounting to 0.03 Å, according to the figure of 6.16 Å reported by Petkov *et al.* (2002 ▸)] is probably related to residual intercalated lithium (with an Li/Mo molar ratio of about 0.06) in the vdW gap after the baking–restacking process. The inter- and intralayer correlated displacement could also be ascribed to this intercalation, due to the reduction of Mo^4+^ and/or to local changes of the local Mo coordination (Petkov *et al.*, 2002 ▸; Prouzet *et al.*, 2003 ▸). The α, β and γ parameters reported in Table 1 ▸ confirm that the structure of our sample is heavily affected by stacking disorder. The deviation from the value γ = 1, corresponding to the ordered sequence of fully eclipsed atoms, points to a partially staggered atomic arrangement in the restacked sample. The average size is calculated as 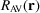 = 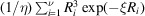 , yielding *R*_AV_ = 51 ± 1 Å. Within the errors of 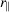 and 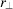 reported in Table 1 ▸, the crystallites are roughly isometric.

## 5. Is the model oversized?

**Figure 4:**
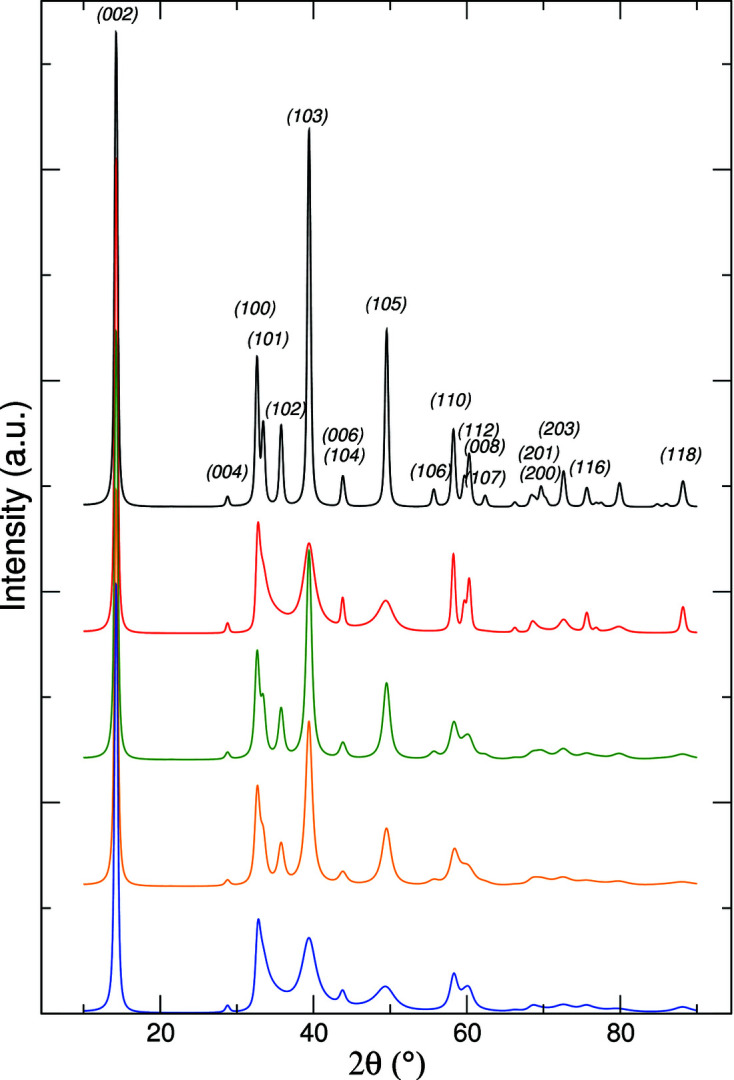
Simulated patterns for exfoliated–restacked MoS_2_. From top to bottom: ordered (black trace), stacking faults only (red), correlated displacement only (green), best fit to the data with correlated displacement only (orange), fitting with both types of disorder (*i.e.* stacking faults and correlated displacement, blue).

We addressed the issue relative to the significance of all the features of the model and, in particular, the question of whether the model is ‘oversized’ as concerns the treatment of structural disorder. In Fig. 4 ▸ we show the simulated patterns relative to (i) the lattice constants and the shape/size parameters reported in Table 1 ▸, no disorder, with Miller index labels; (ii) the same as item (i) plus the stacking fault parameters gathered from Table 1 ▸; (iii) the same as item (i) plus the correlated displacement disorder quantified by the parameters reported in Table 1 ▸; (iv) the fitting to the data with only correlated displacement disorder; (v) the fitted pattern already drawn in Fig. 3 ▸.

Inspection of Fig. 4 ▸ confirms (Kakinoki, 1967 ▸) that stacking faults do not produce broadening of the peaks with Miller indexes 00*l* and *hk*0 or of the reflections with *h* + *k* = 3*n*. On the other hand, the correlated displacement uncertainty determines a progressive broadening of the diffraction peaks, increasing with the modulus of the scattering vector *q*. It is evident that the selective damping of the diffraction peaks induced by stacking faults produces the definite blurring of high-intensity peaks, such as the 102, 103 and 105 peaks, which is not so effectively obtained by the action of correlated displacement only. To further support this analysis, a fitting run assuming no stacking faults and only correlated dis­place­ment was also carried out, to check the possibility that allowing for stacking faults could underestimate the correlated displacement distribution. The fitting, clearly unsatisfactory, confirms that, within the proposed model, both stacking faults and correlated displacement should be allowed.

## 6. Turbostratic arrangement

A limiting case of stacking disorder in TMDC layered structures is turbostratic stacking, proposed since the early papers by Frindt and coworkers (Yang *et al.*, 1991 ▸; Yang & Frindt, 1996 ▸) as a possible tridimensional arrangement, in analogy with turbostratic graphite. In an ideal turbostratic TMDC structure the S–TM–S layers are piled up parallel to one another, but with a completely random relative position. The Frindt group simulations for restacked MoS_2_ were sound, even if not completely suitable for the reported experimental data. Later, the turbostratic arrangement was taken into consideration by Mangelsen *et al.* (2019 ▸) for WS_2_ and by Bekx-Schürmann *et al.* (2020 ▸) for MoS_2_. These authors carried out a PDF analysis of the respective XRD data, allowing for stacking faults and small random displacements of the S–TM–S sandwiches parallel and perpendicular to the basal planes, concluding that both sources of disorder are necessary for a suitable fitting to the data. The question of turbostratic disorder in restacked WS_2_ was addressed also by Petkov *et al.* (2000 ▸) who showed, by PDF analysis, that the nanostructured material undergoes a prismatic-trigonal to distorted-octahedral rearrangement of the tungsten coordination, giving rise to locally different S–W distances; on the basis of this analysis, the turbostratic disorder was ruled out and a distortion within the S–TM–S sandwiches was allowed. Petkov and coworkers also observed that a similar analysis can not easily be extended to MoS_2_, due to the higher instability of the metastable octahedral coordination in this compound.

**Figure 5:**
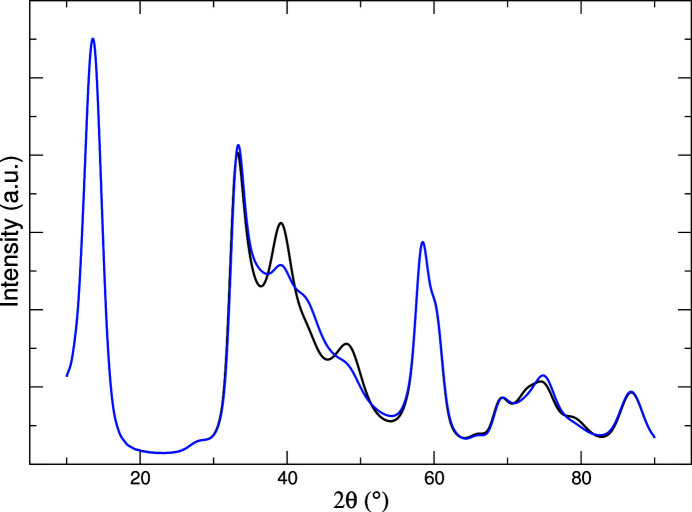
Simulated patterns of MoS_2_ with two combinations of disorder parameters: α = β = 0.27, γ = 0.2 (black trace); α = β = 0.33, γ = 0.0 (blue trace). In both cases, *a* = 3.155, *c* = 6.4 Å.

It is clear that the finer details of preparation can result in quite different samples and, in this respect, all the above-cited analyses are based on specific experimental evidence coming also from spectroscopic data in addition to diffraction techniques. In Fig. 5 ▸ we report the XRD simulation using the lattice parameters from Bekx-Schürmann *et al.* (2020 ▸) and assuming, like these authors, a finite-width distribution in the mutual position of ideal trigonal-prismatic coordinated triple layers. Stacking faults were modulated by trial and error, in order to get a calculated pattern similar to those reported in Fig. 5 of that paper (Bekx-Schürmann *et al.*, 2020 ▸). The most evident discrepancy with the reported data can be observed for the 118 peak, at about 87° 2θ (at 10.76° 2θ using λ = 0.20728 Å), which is not blurred by stacking faults and is only partially blurred by interlayer correlated displacement. As a consequence, we argue that intralayer disorder is effective. It is also worth noting that, with respect to the fitting parameters reported in Table 1 ▸, the same overall pattern shape is obtained with α = β = 0.27, γ = 0.2 and then by reducing, with respect to the refined parameters relative to our sample, the probability of the AbA–BaB sequence corresponding to fully eclipsed atoms along **c**. This trend is even more remarkable in the second simulation reported in Fig. 5, showing the case α = β = 0.33, γ = 0.0, and yielding a profile similar to the one reported in Fig. 3*b* of the paper by Yang *et al.* (1991 ▸).

## 7. Octahedral arrangement

The octahedral coordination of Mo in MoS_2_ was observed as a consequence of intercalation with different guests (Petkov *et al.*, 2002 ▸; Acerce *et al.*, 2015 ▸; Wang *et al.*, 2017 ▸; Stavrou *et al.*, 2022 ▸) or as a result of special preparation techniques (Parilla *et al.*, 2004 ▸; Geng *et al.*, 2017 ▸; Ding *et al.*, 2019 ▸); the variety of reported structural details is probably related to preparation routes and to materials composition.

The possibility of octahedral coordination is investigated in this study by allowing two kinds of S–Mo–S sandwiches with prismatic and octahedral coordination, each one characterized by its own thickness and then by two different spacings, |**c**_P_| and |**c**_O_|, along the direction perpendicular to the S–Mo–S layers. The 12 × 12 transition matrix, an analogue of **P** in equation (1), taking into account the prismatic–prismatic (AbA–AbA-like), the octahedral–octahedral (AbC–AbC), the prismatic–octahedral (AbA–AbC) and the octahedral–prismatic (AbC–AbA) sequences, is shown in equation S1 of the supporting information. It is clear that the overall number of probability parameters involved is too large for a significant refinement and therefore a drastic shortcut was imposed by assuming that the prismatic–octahedral and octahedral–prismatic sequences are governed by a unique parameter (respectively, indicated as η and ε in equation S1). The different spacings, |**c**_P_| and |**c**_O_|, require that the vertical distance between two S–Mo–S layers is a function of the height jump (that is, of the integer *m* in the interatomic distance vector 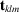 = 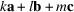 ) and of the path between the terminal layers (Kakinoki & Komura, 1965 ▸); due to the huge number of intermediate step combinations, for large *m* this vertical distance tends asymptotically to 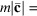 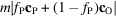 , where 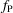 is the fraction of prismatic layers, irrespective of the type of terminal layers.

Fitting of this ‘prismatic–octahedral’ model yields only a marginal improvement, with a decrease of the goodness-of-fit parameter to *R*_P_ = 11.9 with respect to the above-quoted *R*_P_ = 12.6. The refined parameters 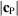 = 6.193 (1) and 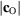 = 6.189 (3) are fairly similar, while the fraction 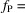 0.8499 (2) denotes, as expected, a large predominance of prismatic coordination. Fig. S1 reports the observed and calculated patterns and Fig. S2 the simulated pattern obtained by excluding the effect of correlated disorder. Some further comments about the occurrence of interatomic distance uncertainty are supported by the simulations reported in Fig. S3.

For a critical appraisal of the prismatic–octahedral model we highlight the following:

(i) The assumption that the S–Mo–S sandwiches are thoroughly prismatic or octahedral is clearly very strong and, taking into account that the octahedral arrangement is locally induced by the intercalated lithium ions (see *e.g.* Chhowalla *et al.*, 2013 ▸), it should be related to a lithium segregation in some of the vdW gaps, rather than a sparse distribution of lithiums.

(ii) This latter distribution of intercalant, producing local perturbations in the S–Mo–S sandwiches, looks to be more realistic, and could also explain why correlated atomic displacements (see Fig. S2) are still necessary to get a good fitting.

(iii) Partial substitution of sulfur with oxygen, producing MoS_2−*x*_O_*x*_ compositions, can be obtained by different synthesis routes involving heat treatments in an oxygen-rich environment (Lince *et al.*, 1995 ▸; Tang *et al.*, 2020 ▸). For relatively thick (∼6 µm) films (Lince *et al.*, 1995 ▸), the materials obtained in this way showed reduced cell constants with respect to pure MoS_2_ and noticeable structural disorder as well. However, considering the presence of residual lithium detected by the chemical analysis and the fact that the restacking procedure was carried out under vacuum, we are inclined to attribute the observed correlated displacement in our sample to the reduction of Mo by intercalated Li (Petkov *et al.*, 2002 ▸; Prouzet *et al.*, 2003 ▸), giving rise to local octahedral rearrangement of molybdenum coordination and to a structural distortion propagating to the neighbouring structural units.

(iv) Overall, it can be concluded that the model presented in Sections 2 and 4 is able to give a detailed picture of the average structure of the investigated material. The prismatic–octahedral model described in this section does not produce a significant improvement; for a thorough structural characterization, integration of the XRD long-range analysis with local techniques such as PDF and/or X-ray absorption spectroscopy should be suitable.

## 8. Conclusions

The XRD patterns reported in the literature and relating to nanosized trigonal-prismatic coordinated TMDCs seem to agree that the main sources of structural disorder are both intralayer and interlayer, while ideal turbostratic disorder should be ruled out. The ‘prismatic’ model presented in this paper treats the correlated displacement of atoms within the formalism of the ‘ideal paracrystal’ (Hosemann, 1951 ▸). Accordingly, the width of the Gaussian distribution governing the mutual displacement of atoms is a linear function of the distance. It is likely that this uncertainty, even if roughly effective in accounting for the quoted experimental evidence, could be more finely modelled. In particular, one could allow for different coordination, such as the distorted octahedral coordination proposed by Petkov *et al.* (2000 ▸), which could be at the origin of a correlated displacement ranging beyond the first neighbours. In the case of the sample investigated by us, residual lithium surviving in the structure after baking–restacking of the monolayer dispersion could influence either the local metal coordination or the stacking sequence of triple layers, or both. Also the ‘prismatic–octahedral’ model described in Section 7 needs correlated displacement in order for a good fit to the data. This fact, and the only marginal improvement obtained with respect to the ‘prismatic’ model, induced us to conclude that, rather than sequences of all-prismatic and all-octahedral sandwiches, intercalated lithium ions determine the above-quoted perturbation of the prismatic coordination of molybdenum.

Stacking faults are necessary to obtain satisfactory data fittings. This kind of disorder has been recognized by several other authors (just to cite the most recent papers: Niefind *et al.*, 2015 ▸; Bekx-Schürmann *et al.*, 2020 ▸; Sanikop & Sudakar, 2020 ▸), but, to our knowledge, this is the first attempt to quantify it on statistical grounds. The model described in Section 2 can be useful for the structural analysis of prismatic TMDCs such as WS_2_ and the respective selenides of Mo and W. The extension to octahedral TMDCs is also straightforward.

## Supplementary Material

Supporting information. DOI: 10.1107/S1600576723001589/iu5037sup1.pdf

## Figures and Tables

**Table 1:** Refined parameters and uncertainties relative to the fitting of the model to the XRD data of the exfoliated–restacked sample of MoS_2_ α, β and γ are defined in equation (1). δ is not a fitting parameter, as it is bound to the condition that the sum of the rows of a stochastic matrix equals 1. 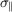 and 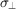 are defined in equations 5(*a*)[Disp-formula fd5a]–5(*b*)[Disp-formula fd5b]. ξ, 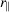 and 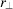 are defined in equation (3) and related comments. *a* and *c* are defined in the comments to equation (2). The goodness of fit was evaluated as 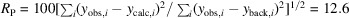 .

α	β	γ	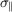 (Å)	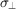 (Å)	ξ (Å^−1^)	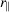 (Å)	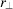 (Å)	*a* (Å)	*c* (Å)
0.37 (1)	0.16 (1)	0.36 (3)	0.0023 (2)	0.0037 (3)	0.057 (1)	142 (5)	151 (3)	3.162 (1)	6.190 (2)
