# Supplementary material for: Modelling the structural disorder in trigonal-prismatic coordinated transition metal dichalcogenides
Source: J Appl Crystallogr. 2023 Mar 30;56(Pt 2):502–9. doi: 10.1107/S1600576723001589 (PMC10077858; doi:10.1107/S1600576723001589)
Supplement: Supplementary file 1 [file j-56-00502-sup1.pdf]

JOURNAL OF APPLIED CRYSTALLOGRAPHY

Volume 56 (2023)

Supporting information for article:

Modeling the structural disorder in trigonal-prismatic coordinated transition metal dichalcogenides

Federica Ursi, Simone Virga, Candida Pipitone, Alessandra Sanson, Alessandro Longo, Francesco Giannici and Antonino Martorana

Supporting information

$$
\mathbf{P} = \begin{bmatrix}
 & AbC & BcB & CaC & AcA & BaB & CbC & AbC & BcA & CaB & AcB & BaC & CbA \\
AbC & 0 & \alpha & \beta & 0 & \gamma & \delta & 0 & \eta & \eta & 0 & \eta & \eta \\
BcB & \beta & 0 & \alpha & \delta & 0 & \gamma & \eta & 0 & \eta & \eta & 0 & \eta \\
CaC & \alpha & \beta & 0 & \gamma & \delta & 0 & \eta & \eta & 0 & \eta & \eta & 0 \\
AcA & 0 & \delta & \gamma & 0 & \beta & \alpha & 0 & \eta & \eta & 0 & \eta & \eta \\
BaB & \gamma & 0 & \delta & \alpha & 0 & \beta & \eta & 0 & \eta & \eta & 0 & \eta \\
CbC & \delta & \gamma & 0 & \beta & \alpha & 0 & \eta & \eta & 0 & \eta & \eta & 0 \\
AbC & \varepsilon & \varepsilon & 0 & \varepsilon & \varepsilon & 0 & \phi & \chi & 0 & \psi & \omega & 0 \\
BcA & 0 & \varepsilon & \varepsilon & 0 & \varepsilon & \varepsilon & 0 & \phi & \chi & 0 & \psi & \omega \\
CaB & \varepsilon & 0 & \varepsilon & \varepsilon & 0 & \varepsilon & \chi & 0 & \phi & \omega & 0 & \psi \\
AcB & \varepsilon & 0 & \varepsilon & \varepsilon & 0 & \varepsilon & \psi & 0 & \omega & \phi & 0 & \chi \\
BaC & \varepsilon & \varepsilon & 0 & \varepsilon & \varepsilon & 0 & \omega & \psi & 0 & \chi & \phi & 0 \\
CbA & 0 & \varepsilon & \varepsilon & 0 & \varepsilon & \varepsilon & 0 & \omega & \psi & 0 & \chi & \phi
\end{bmatrix} \qquad \text{(S1)}
$$

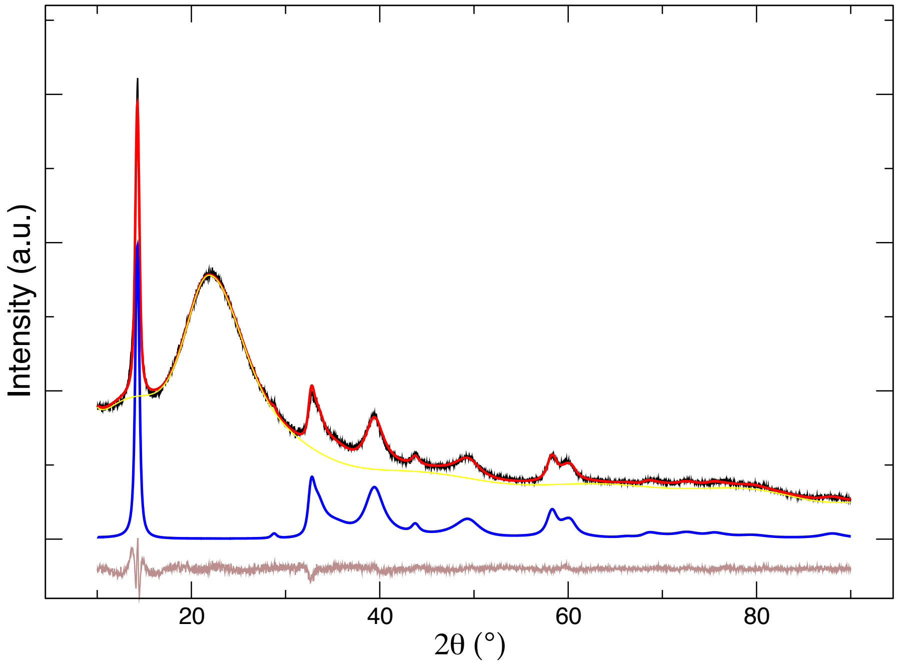

Figure S1 Fitting of the prismatic-octahedral model to the XRD data of the exfoliated-restacked MoS_2 sample. Experimental, black; calculated, red; background, yellow; model, blue; residual, brown.

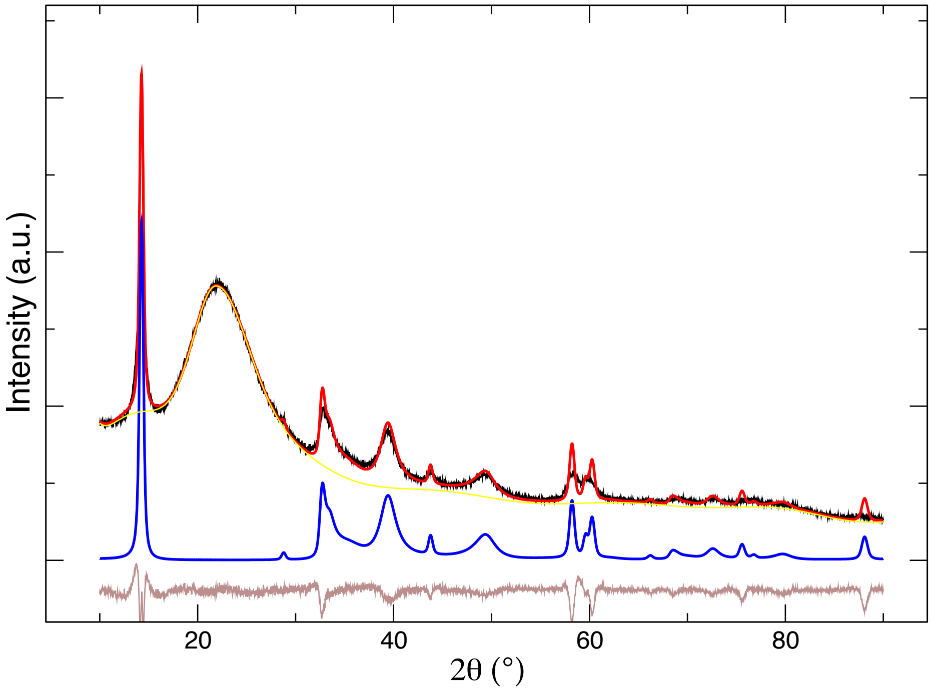

Figure S2 Simulation of the prismatic-octahedral model to the XRD data of the exfoliated-restacked MoS_2 sample. Experimental, black; calculated, red; background, yellow; model, blue; residual, brown. The correlated interatomic distance uncertainty is eliminated.

Figure S2 shows that the presence of two different S-Mo-S thicknesses is not the main source of interatomic distance uncertainty with consequent blurring of the high-angle lines. This evidence was confirmed by simulations involving a small (f_P=0.5) frequency of prismatic layers and a quite large difference between prismatic and octahedral thicknesses ($|\mathbf{c}_P|$=6.19 Å and $|\mathbf{c}_O|$=6.23 Å): figure S3 shows that the components of the total XRD pattern corresponding to different height jumps between m-neighbouring sandwiches (that is, the value of the integer m in the interatomic distance vector $\mathbf{t}_{klm} = k\mathbf{a} + l\mathbf{b} + m\mathbf{c}$) remain sharp despite the large difference between the prismatic and octahedral thicknesses if the interatomic distance correlated uncertainty is not considered (blue traces); on the contrary, if this source of disorder is considered (red traces), the blurring of the intensity components increases as a function of the scattering angle and of the distance (*i.e.* at increasing m) between the S-Mo-S sandwiches. On the contrary, the effect of blurring the high-angle lines determined by the presence of two different thicknesses decreases at increasing m, because at high m values the spacing between m-

neighbouring layers tends asymptotically to the unique $m|\bar{\mathbf{c}}| = m|f_P\mathbf{c}_P + (1 - f_P)\mathbf{c}_O|$ value, no matter of the terminal layers.

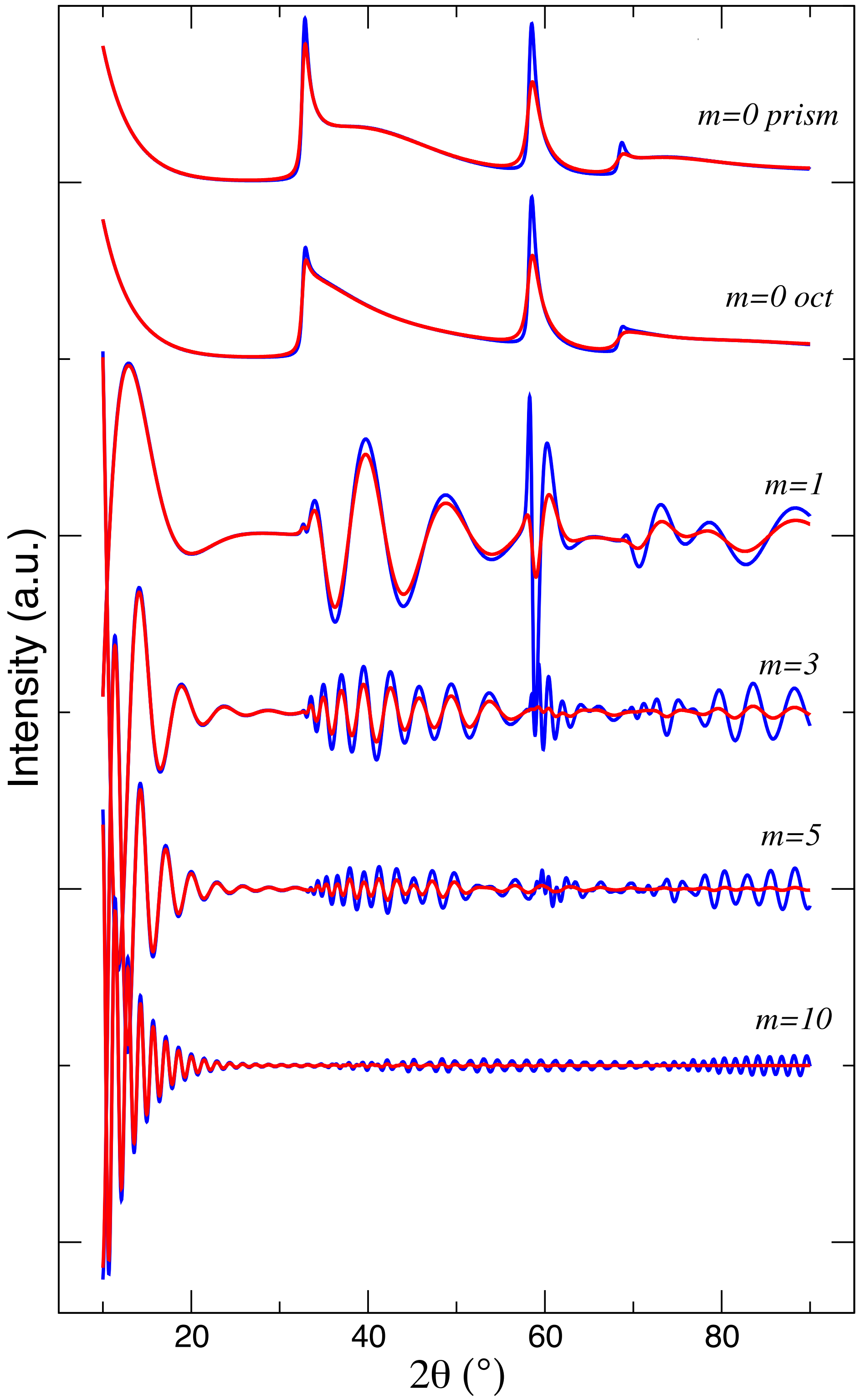

Figure S3 Components of the total XRD pattern corresponding to different height jumps (see text) between S-Mo-S sandwiches. Blue traces, no interatomic distance correlated uncertainty; red traces, with correlated uncertainty. For *m*=0 (self-scattering of the S-Mo-S units) the distinct contributions of prismatic and octahedral sandwiches are drawn.
